# A simplified Fazekas scale for efficient assessment of white matter hyperintensities

**DOI:** 10.3389/fneur.2026.1855460

**Published:** 2026-10-05

**Authors:** Peng Liu, Songguo Liu, Chuanna Xu, Ming Zhu, Xue Liu, Xin Zhao, Bentao Wei, Qiang Li, Guang Han

**Affiliations:** Department of Radiology, Linyi Hospital of Traditional Chinese Medicine, Linyi, China

**Keywords:** cerebral small vessel disease, clinical assessment, simplified Fazekas scale, visual rating scale, white matter hyperintensities

## Abstract

**Background:**

White matter hyperintensities (WMH) are key neuroimaging markers of cerebral small vessel disease (CSVD) and are associated with neuropsychiatric symptoms in older adults. Although the Fazekas visual rating scale is widely used in research, its clinical adoption remains limited. Therefore, we developed a simplified Fazekas visual rating scale (sFazekas) for overall WMH assessment to facilitate clinical application.

**Methods:**

We randomly selected 900 participants from three MRI scanners and evaluated the reliability and scoring efficiency of sFazekas versus the Fazekas scale. Then, all participants were grouped according to the sFazekas scores, and intergroup differences among the different scores were analyzed. Next, we examined correlations among the sFazekas, the Fazekas, and WMH volume. Finally, we explored associations between the sFazekas and vascular risk factors related to CSVD.

**Results:**

The sFazekas demonstrated excellent inter-rater reliability (*κ* = 0.851) and required significantly less time to complete than the conventional Fazekas (*p* < 0.01). Significant differences in age, WMH volume, and vascular risk factors were observed across sFazekas score groups (*p* < 0.01). The sFazekas scores were strongly correlated with Fazekas scores for both deep and periventricular white matter, as well as with WMH volume (*p* < 0.001). Ordinal logistic regression indicated that age, hypertension, diabetes mellitus, and a history of stroke were independent predictors of higher sFazekas scores (*p* < 0.001).

**Conclusion:**

The simplified Fazekas visual rating scale (sFazekas) demonstrates reliability comparable to the conventional Fazekas scale, with the practical advantage of shorter scoring time, suggesting its potential utility in the clinical assessment of overall white matter hyperintensity burden in patients with cerebral small vessel disease.

## 1 Introduction

Cerebral small vessel disease (CSVD) encompasses a spectrum of imaging, pathological, and clinical syndromes arising from structural and functional abnormalities of the brain’s small arteries, arterioles, venules, and capillaries (1). White matter hyperintensities (WMH), the key imaging biomarker of CSVD, appear on MRI as regions of increased signal intensity on T2-weighted and fluid-attenuated inversion recovery (FLAIR) sequences. These lesions typically exhibit a symmetrical distribution and are predominantly located around the ventricles or within the subcortical white matter (2). A growing body of evidence has demonstrated that WMH are closely linked to cognitive impairment, motor deficits, and various neuropsychiatric symptoms (3, 4). Therefore, WMH should not be viewed merely as an inevitable or “silent” manifestation of brain aging.

Visual rating remains a key approach for evaluating the severity of WMH. Among the various visual rating systems developed for WMH evaluation, the Fazekas visual rating scale (Fazekas scale) is the most widely used. Introduced by Fazekas et al. (5), this scale differentiates WMH into periventricular white matter (PVWM) and deep white matter (DWM) based on anatomical location, and grades lesion severity from 0 to 3 according to their morphological appearance and degree of confluence. The Fazekas scale is highly regarded and commonly applied in research settings (6).

However, the anatomical definitions of the subregions in the conventional Fazekas scale are not entirely consistent across studies, and pathological investigations have indicated that the boundary between DWM and PVWM is often indistinct, potentially increasing the complexity and subjectivity of region-specific assessment (7–11). Moreover, in clinical settings, the assessment of WMH focuses more on overall burden, whereas separate assessment of different anatomical regions may make it less straightforward to describe overall WMH burden. These limitations highlight the need for a simple and reproducible approach that can provide a unified assessment of overall WMH burden without requiring region-specific assessment.

Therefore, this study, conducted in real-world clinical settings and building upon the Fazekas scale and previous modifications, aimed to: (i) establish a simplified Fazekas visual rating scale (sFazekas scale) for overall assessment; (ii) evaluate the inter-rater reliability of sFazekas scale; (iii) explore the correlation between the sFazekas scale and WMH volume; and (iv) explore the association between the sFazekas scale and vascular risk factors and further evaluate its potential utility in the clinical assessment of overall WMH burden in patients with CSVD.

## 2 Materials and methods

### 2.1 Participants

We conducted a retrospective study based on a hospital-based cohort collected during 2024. All participants were first screened according to the predefined inclusion and exclusion criteria during the 1-year study period. Eligible participants were then stratified by MRI scanner: GE Discovery MR750 3.0 T, GE SIGNA Architect 3.0 T, and uMR588 1.5 T. Within each scanner stratum, 300 patients were randomly selected, yielding a final study sample of 900 participants and ensuring balanced representation across scanners. Inclusion criteria: (i) age ≥50 years; (ii) availability of complete MRI sequences (T1WI and T2 FLAIR); (iii) meeting the STRIVE-1/2 diagnostic criteria; (iv) availability of complete vascular risk factor data, according to the definitions provided by the U.S. Department of Health and Human Services, including hypertension, hyperlipidemia, diabetes mellitus, heart disease, and a history of stroke (12). Exclusion criteria: (i) large cerebral infarction (maximum axial diameter ≥20 mm); (ii) encephalomalacia (maximum diameter >15 mm); (iii) structural intracranial abnormalities (e.g., mass lesions, malformations); (iv) intracranial hemorrhage; (v) Image artifacts. This study was approved by the Ethics Committee.

### 2.2 MRI acquisition

T2 FLAIR images were acquired on the GE Discovery MR750 and GE SIGNA Architect scanners with the following parameters: echo time (TE) = 130 ms, repetition time (TR) = 9,000 ms, slice thickness = 5 mm, and interslice gap = 1 mm. T1-weighted images (T1WI) were obtained with TE = 24 ms, TR = 1750 ms, slice thickness = 5 mm, and interslice gap = 1 mm. On the United Imaging uMR588 scanner, T2 FLAIR images were acquired with TE = 97.24 ms, TR = 8,000 ms, slice thickness = 5 mm, and interslice gap = 1 mm, while T1WI images were obtained with TE = 23.68 ms, TR = 2,300 ms, slice thickness = 5 mm, and interslice gap = 1 mm.

### 2.3 Fazekas and sFazekas scale rating

Prior to conducting ratings using the Fazekas and sFazekas scales, a group meeting was held to integrate and refine the existing Fazekas scale in order to develop the sFazekas scale for a comprehensive overall assessment. Subsequently, all T2 FLAIR images included in the study were independently assessed by two raters, each with over 10 years of neuroimaging experience. The Fazekas scale was applied to two components of white matter, deep and periventricular, yielding Fazekas scores for deep (F-DWM) and periventricular white matter (F-PVWM). For F-DWM, 0 indicates no lesion, 1 represents punctate foci, 2 denotes the beginning confluence of lesions, and 3 corresponds to large confluent lesions. For F-PVWM, 0 indicates no lesion, 1 represents caps or pencil-thin lining, 2 denotes a smooth halo, and 3 corresponds to irregular periventricular signal extending into the deep white matter (5). The sFazekas scale assesses WMH throughout the entire cerebral hemisphere and assigns a score from 0 to 3: 0 indicates no lesions; 1 represents punctate or nodular lesions, or thin-layer lesions; 2 denotes periventricular patchy lesions, periventricular band-like lesions, or confluent nodular lesions in the deep white matter; and 3 corresponds to large or irregular confluent lesions (Figure 1). The scoring does not include the corpus callosum or infratentorial structures. Both raters were blinded to the participants’ clinical information, and all ratings were performed on workstations with identical configurations.

**Figure 1:**
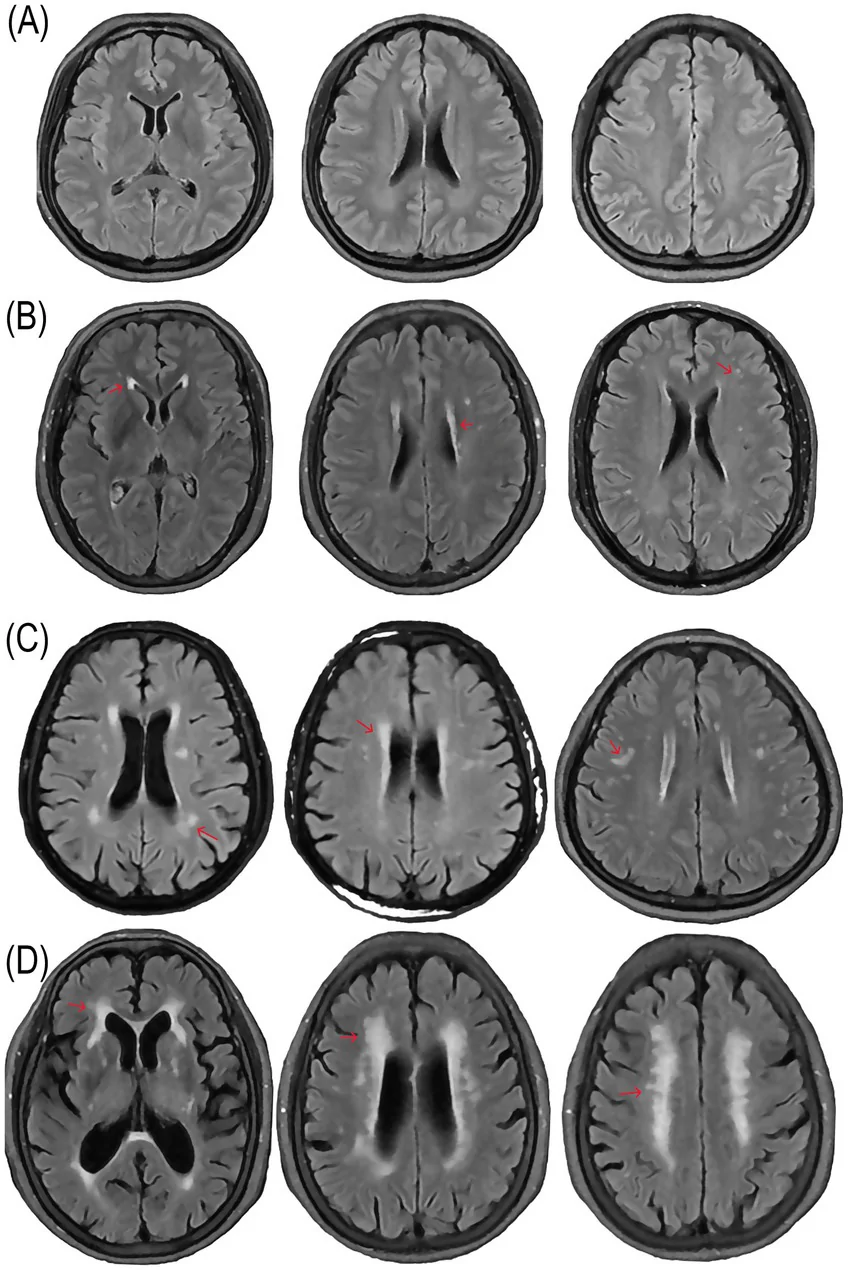
Simplified Fazekas visual rating scale on representative MRI images. Representative MRI images illustrating the Simplified Fazekas visual rating scale (SFazekas), with red arrows indicating lesions. Group **(A)** shows Grade 0 (no lesions); Group **(B)** shows Grade 1 (punctate/nodular or thin-layer lesions); Group **(C)** shows Grade 2 (periventricular patchy lesions, periventricular band-like lesions, or confluent nodular lesions in the deep white matter); Group **(D)** shows Grade 3 (large or irregular confluent lesions).

### 2.4 WMH segmentation and quantification

All participants’ T1-weighted and T2-FLAIR images were nonlinearly registered using the antsMultivariateTemplateConstruction command from ANTs.^1^ Subsequently, automated WMH segmentation was performed using the Sysu_media algorithm (accessed in June 2025), with the co-registered T1-weighted and T2-FLAIR images as input. The Sysu_media algorithm was the winning solution in the MICCAI 2017 WMH Segmentation Challenge and was validated on the challenge’s independent test set, achieving a mean Dice similarity coefficient of 0.80 and a mean recall of 0.84 (13). This algorithm is a deep learning-based ensemble model of fully convolutional networks, employing a two-channel U-Net architecture comprising a downsampling path for reducing spatial dimensions and an upsampling path for reconstructing segmentation maps. Segmentation was performed using the Docker image of this algorithm^2^ on Ubuntu 20.04. The source code is available at hongweilibran/wmh_ibbmTum. Further information, including the training data and test-set results, is publicly available at https://wmh.isi.uu.nl/results/. WMH volume was calculated from the segmented masks using custom Python scripts (PyCharm 2024.1.4, Python 3.12), based on voxel dimensions derived from the image header.

### 2.5 Statistical analysis

All statistical analyses were conducted using SPSS (version 27.0), RStudio (version 2024.04.2), and PyCharm (version 2024.1.4). A two-sided *p*-value < 0.05 was considered statistically significant.

#### 2.5.1 Study population characteristics

Descriptive statistical analyses were performed for the participants’ demographic and clinical characteristics, including age, sex, vascular risk factors, Fazekas score, sFazekas score, and WMH volume (mL). Continuous variables were expressed as mean ± standard deviation (Mean ± SD). Categorical variables were presented as frequency (n) and percentage (%). Ordinal variables were summarized as median with interquartile range [Median (Q1, Q3)].

#### 2.5.2 Inter-rater reliability and scoring efficiency

The consistency of the ratings between two evaluators was assessed using the weighted Cohen’s Kappa coefficient (*κ*) with quadratic weights. The κ value and its 95% confidence interval (CI) were reported. The level of agreement was categorized into five grades: excellent (*κ* > 0.80), good (*κ* = 0.61–0.80), moderate (*κ* = 0.41–0.60), fair (*κ* = 0.21–0.40), and poor (*κ* ≤ 0.20) (14). Inter-rater agreement was assessed using quadratic weighted Cohen’s *κ*. The agreement of the sFazekas scale was compared separately with that of F-PVWM and F-DWM by calculating the difference in *κ* (Δ*κ* = *κ*_sFazekas − *κ*_F-PVWM/DWM). Non-parametric bootstrap resampling with 5,000 iterations was used to estimate the 95% confidence intervals and two-sided *p* values for Δ*κ*.

To evaluate the scoring efficiency of the sFazekas scale compared with the Fazekas scale, a subset of 300 participants was pseudo-randomly selected from the cohort, with 100 scanned on each MRI scanner model. The same rater assessed this cohort using the sFazekas scale and the conventional Fazekas scale, with a 2-week interval between the assessments using the two scales, and the scoring time for each case was recorded. The scoring time was independently and synchronously recorded by two observers who were not involved in the scoring process using primary and backup digital stopwatches, with a recording precision of 0.01 s. Timing started when the rater indicated “start scoring” and ended when the rater indicated “end scoring.” When the discrepancy between the primary and backup timers was ≤0.5 s, the value recorded by the primary timer was used. When the discrepancy was >0.5 s, the timing result for that case was excluded from the analysis, and the case was placed into a new assessment queue. After 2 weeks, the case was rescored using the corresponding scoring scale, and the scoring time was recorded again. A paired *t*-test was performed to compare rating efficiency between the two scales, with statistical significance set at *p* < 0.05.

#### 2.5.3 Analysis of intergroup differences

All participants were grouped according to the sFazekas scores, and intergroup differences among the different scores were analyzed. For continuous variables, the Shapiro–Wilk test was first used to assess normality, and Levene’s test was employed to evaluate the homogeneity of variance across groups. Since the distributions of age and WMH volume were skewed, the Kruskal–Wallis H test was used to compare age and WMH volume across different sFazekas scores. When the overall test was significant, Dunn’s *post hoc* test was further conducted, with the Holm method applied for multiple comparisons correction. For categorical variables (sex, hypertension, hyperlipidemia, diabetes, heart disease, and a history of stroke), Pearson’s chi-square test was used to assess differences in distribution among sFazekas scores. Overall differences among groups were evaluated using the chi-square test. When the overall comparison was statistically significant, pairwise comparisons were conducted. Fisher’s exact test was used for 2 × 2 contingency tables when any expected cell count was below 5. *p* values for pairwise comparisons were adjusted for multiple comparisons using the Bonferroni method.

#### 2.5.4 Correlation analysis

Since both the sFazekas and Fazekas (F-DWM, F-PVWM) scores are ordinal variables, and the WMH volume data are not normally distributed, Spearman’s rank correlation coefficient (*ρ*) was used to assess the correlations among sFazekas, F-DWM, F-PVWM, and WMH volume. Correlation coefficients (*ρ*) and significance levels (*p* values) are reported.

#### 2.5.5 Ordinal logistic regression analysis

We conducted separate ordinal logistic regression analyses with the sFazekas, F-DWM, and F-PVWM scores as dependent variables. The independent variables were sex, hypertension, hyperlipidemia, diabetes, heart disease, and a history of stroke, all coded as dichotomous variables, as well as age, which was treated as a continuous variable. Model parameters were estimated using the maximum likelihood method. For each independent variable, odds ratios with corresponding 95% confidence intervals were reported. All statistical tests were two-sided, with a significance level set at *α* = 0.05. The Brant test was used to assess the proportional-odds assumption. In cases of violation with a significant predictor, a partial proportional odds model was used to allow threshold-specific effects.

## 3 Results

### 3.1 Participant characteristics

Table 1 shows the characteristics of the study participants. A total of 900 participants were enrolled in this study, including 398 males (44.2%) and 502 females (55.8%), with a mean age of 62.98 ± 8.86 years. The median (interquartile range) scores for the sFazekas, F-PVWM, and F-DWM were 1 (1–2), 1 (0–2), and 1 (1–1), respectively. The volume of WMH was 4.48 ± 6.60 mL. Additionally, the numbers of participants in each sFazekas grade were 64 (7.1%) for grade 0, 597 (66.3%) for grade 1, 150 (16.7%) for grade 2, and 89 (9.9%) for grade 3. For F-DWM, the corresponding numbers were 75 (8.3%), 617 (68.6%), 137 (15.2%), and 71 (7.9%), respectively. For F-PVWM, the corresponding numbers were 326 (36.2%), 345 (38.3%), 143 (15.9%), and 86 (9.6%), respectively.

**Table 1:** Participants’ characteristics.

Characteristic	All (*N* = 900)	GE_750 (*N* = 300)	GE_Architect (*N* = 300)	uMR588 (*N* = 300)
Demographics				
Age (years)	62.98 ± 8.86	62.61 ± 8.72	62.80 ± 8.69	63.53 ± 9.16
Female, *N* (%)	502(55.8%)	175(58.3%)	159(53.0%)	168(56.0%)
Vascular risk factors				
Hypertension, *N* (%)	405 (45.0%)	145 (48.3%)	138 (46.0%)	122 (40.7%)
Hyperlipidemia, *N* (%)	79 (8.8%)	22 (7.3%)	37 (12.3%)	20 (6.7%)
Diabetes, *N* (%)	216 (24.0%)	72 (24.0%)	78 (26.0%)	66 (22.0%)
Heart disease, *N* (%)	160 (17.8%)	55 (18.3%)	60 (20.0%)	45 (15.0%)
History of stroke, *N* (%)	131 (14.6%)	53 (17.7%)	40 (13.3%)	38 (12.7%)
Imaging characteristics				
F-PVWM, median, IQR	1 (0–2)	1 (0–2)	1 (0–1)	1 (0–2)
F-DWM, median, IQR	1 (1–1)	1 (1–2)	1 (1–1)	1 (1–1)
sFazekas, median, IQR	1 (1–2)	1 (1–2)	1 (1–1)	1 (1–2)
WMH-Volume (mL)	4.48 ± 6.60	5.08 ± 7.36	4.25 ± 5.62	4.10 ± 6.68

sFazekas, Simplified Fazekas visual rating scale; F-PVWM, Fazekas scale for periventricular white matter; F-DWM, Fazekas scale for deep white matter; WMH, White matter hyperintensities.

### 3.2 Inter-rater reliability and scoring efficiency

As shown in Table 2, the sFazekas scale, F-PVWM and F-DWM demonstrated excellent inter-rater reliability, with *κ* values of 0.851, 0.816, and 0.839, respectively. Compared with F-PVWM, the sFazekas scale showed a Δ*κ* of 0.035 (95% CI: 0.007–0.063, *p* = 0.014); compared with F-DWM, the Δ*κ* was 0.012 (95% CI: −0.007–0.032, *p* = 0.239). Moreover, Figure 2 shows the sFazekas scale required significantly less time to complete than the conventional Fazekas scale (9.02 ± 1.99 s vs. 12.95 ± 2.90s, *p* < 0.01).

**Table 2:** Inter-rater agreement of sFazekas and Fazekas scales.

Scales	Machine	Weighted Cohen’s Kappa	*p*-value
sFazekas	All	0.851	<0.001
	GE_750	0.876	<0.001
	GE_Architect	0.814	<0.001
	uMR588	0.855	<0.001
F-PVWM	All	0.816	<0.001
	GE_750	0.803	<0.001
	GE_Architect	0.757	<0.001
	uMR588	0.878	<0.001
F-DWM	All	0.839	<0.001
	GE_750	0.867	<0.001
	GE_Architect	0.816	<0.001
	uMR588	0.831	<0.001

sFazekas, Simplified Fazekas visual rating scale; F-PVWM, Fazekas scale for periventricular white matter; F-DWM, Fazekas scale for deep white matter; WMH, White matter hyperintensities.

**Figure 2:**
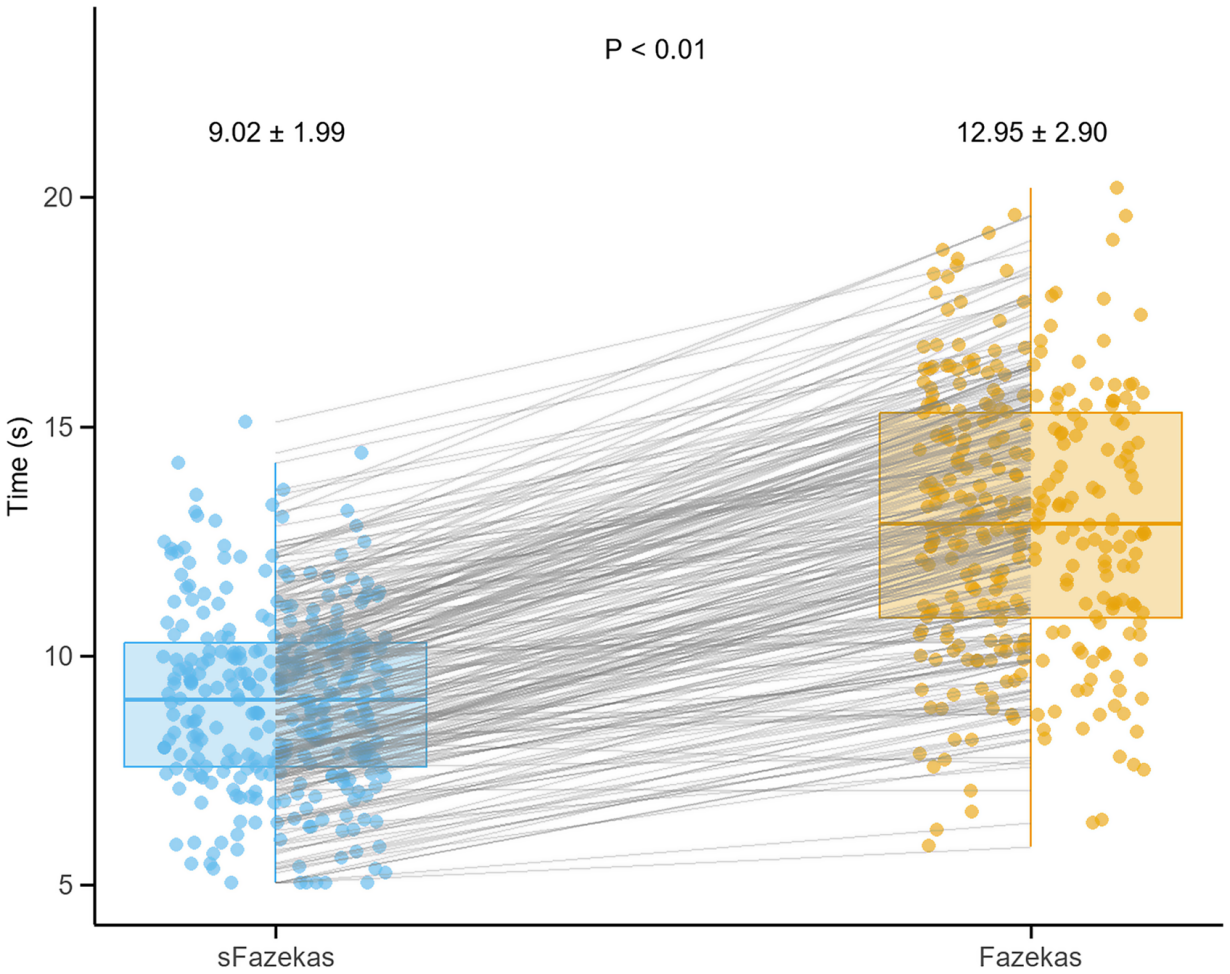
Comparison of scoring completion time between sFazekas and Fazekas scores. Boxplots show the median and interquartile range; jittered points indicate individual measurements; gray lines connect 80% of paired data points. The mean ± SD for each group is shown above the boxes. Colors indicate groups: sFazekas (blue) and Fazekas (orange). A paired *t*-test revealed a significant difference (*p* < 0.01).

### 3.3 Analysis of intergroup differences

The results of the Kruskal–Wallis H test indicated significant differences in age across different sFazekas scores (*H* = 199.909, *p* < 0.001). Further *post hoc* analysis using Dunn’s test revealed that all pairwise comparisons between groups were statistically significant (adjusted *p* < 0.001). Similarly, WMH volume differed significantly among different sFazekas scores (*H* = 477.859, *p* < 0.001). Subsequent Dunn’s *post hoc* tests showed that WMH volume differences between all groups were statistically significant (adjusted *p* < 0.001). WMH volume progressively increases with higher sFazekas scores (Figure 3).

**Figure 3:**
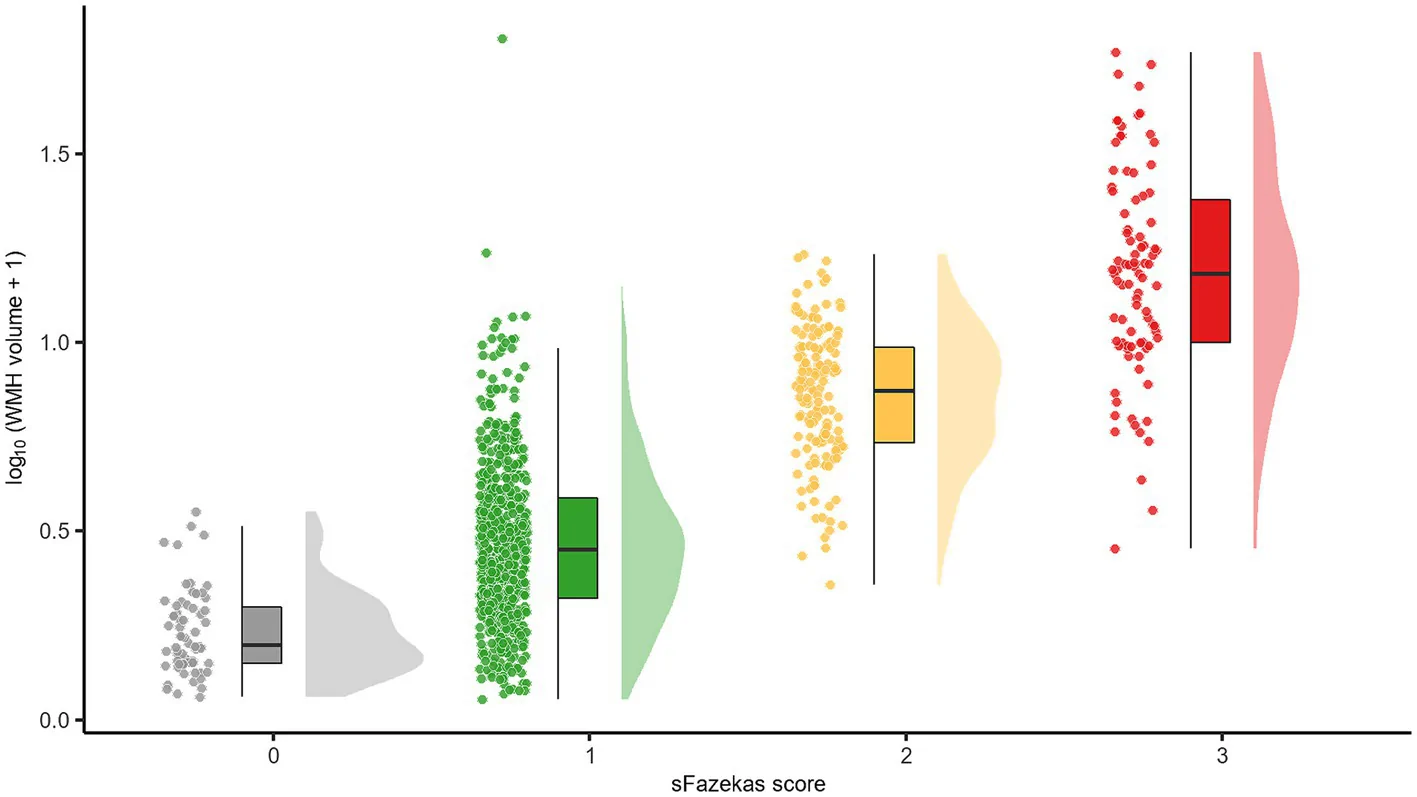
Log10-transformed WMH volume (log10[WMH_V + 1]) across sFazekas scores 0–3. Half-violin plots depict data density, half-boxplots indicate median and interquartile range, and half-scatter plots show individual values. Colors denote sFazekas scores: gray (0), green (1), orange-yellow (2), red (3). Higher values correspond to greater lesion burden with increasing sFazekas score. sFazekas, Simplified Fazekas visual rating scale; WMH, White matter hyperintensities.

Table 3 presents the results of the Pearson chi-square test, showing significant differences in the distributions of hypertension, hyperlipidemia, diabetes, heart disease, and a history of stroke across the different sFazekas score (*p* < 0.05). However, in pairwise comparisons, some between-group differences did not reach statistical significance. No significant differences in sex distribution were observed among the different sFazekas score (*p* > 0.05).

**Table 3:** Chi-square tests of sex and vascular risk factors across sFazekas scores.

Variable	*χ*^2^ value	*p*-value	Score	Pairwise_prop_test_p	Fisher_p_value*
Sex	2.300	*p* = 0.513	0 vs. 1	–	–
			0 vs. 2	–	–
			0 vs. 3	–	–
			1 vs. 2	–	–
			1 vs. 3	–	–
			2 vs. 3	–	–
Hypertension	38.126	*p* < 0.001	0 vs. 1	*p* = 1.000	–
			0 vs. 2	*p* = 0.005	–
			0 vs. 3	*p* < 0.001	–
			1 vs. 2	*p* = 0.001	–
			1 vs. 3	*p* < 0.001	–
			2 vs. 3	*p* = 0.954	–
Hyperlipidemia	35.653	*p* < 0.001	0 vs. 1	–	*p* = 0.098
			0 vs. 2	–	*p* = 0.218
			0 vs. 3	*p* < 0.001	–
			1 vs. 2	*p* = 1.000	–
			1 vs. 3	*p* < 0.001	–
			2 vs. 3	*p* = 0.002	–
Diabetes	26.933	*p* < 0.001	0 vs. 1	*p* = 1.000	–
			0 vs. 2	*p* = 0.018	–
			0 vs. 3	p = 0.018	–
			1 vs. 2	*p* = 0.001	–
			1 vs. 3	*p* = 0.004	–
			2 vs. 3	*p* = 1.000	–
Heart disease	10.631	*p* = 0.014	0 vs. 1	*p* = 1.000	–
			0 vs. 2	*p* = 1.000	–
			0 vs. 3	*p* = 1.000	–
			1 vs. 2	*p* = 0.053	–
			1 vs. 3	*p* = 0.217	–
			2 vs. 3	*p* = 1.000	–
History of stroke	56.092	*p* < 0.001	0 vs. 1	*p* = 0.236	–
			0 vs. 2	*p* < 0.001	–
			0 vs. 3	*p* < 0.001	–
			1 vs. 2	*p* < 0.001	–
			1 vs. 3	*p* < 0.001	–
			2 vs. 3	*p* = 1.000	–

*Fisher’s exact test was used for hyperlipidemia in the 0 vs. 1 and 0 vs. 2 comparisons because the expected cell count was <5. All pairwise comparison *p* values were adjusted using the Bonferroni method. sFazekas, simplified Fazekas visual rating scale.

### 3.4 Correlation analysis

The sFazekas score demonstrated strong correlations with the F-PVWM (*ρ* = 0.739, 95% CI: 0.706–0.770, *p* < 0.001) and F- DWM (*ρ* = 0.907, 95% CI: 0.882–0.930, *p* < 0.001), as well as with the total WMH volume (*ρ* = 0.727, 95% CI: 0.695–0.757, *p* < 0.001). The F-PVWM showed a strong correlation with total WMH volume (*ρ* = 0.784, 95% CI: 0.753–0.811, *p* < 0.001), as did F-DWM (*ρ* = 0.682, 95% CI: 0.645–0.716, *p* < 0.001).

### 3.5 Regression analysis

Table 4 presents the results of ordinal logistic regression analyses examining the associations of age, sex, and vascular risk factors with sFazekas, F-PVWM, and F-DWM scores. For the sFazekas scale, the proportional odds assumption was satisfied. Age (OR = 1.134, 95% CI: 1.113–1.155, *p* < 0.001), hypertension (OR = 1.864, *p* < 0.001), diabetes (OR = 1.517, *p* = 0.020), and history of stroke (OR = 2.353, *p* < 0.001) were significant predictors of higher scores, whereas sex, hyperlipidemia, and heart disease were not. For F-PVWM, a partial proportional odds model was used because the proportional odds assumption was violated for age. Age was significantly associated with F-PVWM across all three thresholds, with ORs ranging from 0.850 to 0.901 (all *p* < 0.001). Hypertension, diabetes, and history of stroke were also significant predictors, whereas sex, hyperlipidemia, and heart disease were not. For F-DWM, the proportional odds assumption was satisfied. Age, sex, hypertension, hyperlipidemia, and history of stroke were significantly associated with higher F-DWM scores, whereas diabetes and heart disease were not. Overall, age, hypertension, and history of stroke were significantly associated with WMH severity across all three WMH scales, whereas diabetes and hyperlipidemia showed scale-specific associations. The threshold-specific associations of age with F-PVWM supported the use of a partial proportional odds model.

**Table 4:** Ordinal logistic regression models evaluating the associations of age, sex, and vascular risk factors with sFazekas and Fazekas (F-DWM and F-PVWM) scores.

Scale	Variable	OR (95% CI)	*p*-value
sFazekas	Age	1.134 (1.113–1.155)	<0.001
	Sex	0.956 (0.715–1.278)	0.763
	Hypertension	1.864 (1.373–2.539)	<0.001
	Hyperlipidemia	1.657 (0.988–2.769)	0.055
	Diabetes	1.517 (1.068–2.155)	0.020
	Heart disease	0.817 (0.556–1.194)	0.299
	History of stroke	2.353 (1.588–3.483)	<0.001
F-PVWM	Age (0 vs. ≥ 1)*	0.901 (0.883–0.920)	< 0.001
	Age (≤1 vs. ≥ 2)*	0.874 (0.856–0.893)	< 0.001
	Age (≤2 vs. 3)*	0.850 (0.823–0.878)	< 0.001
	Sex	0.951 (0.739–1.224)	0.697
	Hypertension	0.633 (0.487–0.823)	<0.001
	Hyperlipidemia	0.810 (0.516–1.270)	0.358
	Diabetes	0.719 (0.529–0.979)	0.036
	Heart disease	1.219 (0.871–1.704)	0.248
	History of stroke	0.584 (0.406–0.840)	0.004
F-DWM	Age	1.125 (1.105–1.147)	<0.001
	Sex	0.731 (0.544–0.980)	0.037
	Hypertension	1.849 (1.356–2.532)	<0.001
	Hyperlipidemia	1.946 (1.164–3.245)	0.011
	Diabetes	1.300 (0.910–1.857)	0.149
	Heart disease	0.785 (0.532–1.154)	0.220
	History of stroke	2.226 (1.488–3.324)	<0.001

*The proportional-odds assumption was violated for age (Brant test, *p* ≈ 0.001); therefore, a partial proportional odds model was applied, allowing the effect of age to vary across thresholds. sFazekas, Simplified Fazekas visual rating scale; F-PVWM, Fazekas scale for periventricular white matter; F-DWM, Fazekas scale for deep white matter.

## 4 Discussion

In this study, we integrated and optimized the existing Fazekas scale in real-world clinical settings, resulting in the establishment of a simplified Fazekas visual rating scale (sFazekas) for overall assessment. The simplified Fazekas visual rating scale (sFazekas) demonstrates reliability comparable to the conventional Fazekas scale, with a shorter scoring time, and correlated strongly with WMH burden, supporting its potential as a rapid and practical tool for clinical evaluation.

The Fazekas scale has long been widely used in research on WMH and remains an important tool for the imaging assessment of CSVD. Currently, several studies have attempted to provide an overall assessment of WMH based on the Fazekas scale. Some methods quantify the overall burden by simply summing the scores of the two regions (15); nevertheless, this approach still suffers from the inherent limitations of region-specific assessments. Other studies have classified lesions according to their diameters (16, 17), which enhances the precision of assessment but increases the time required for clinical application. Additionally, certain morphology-based approaches focus predominantly on deep white matter regions (18), making it challenging to capture periventricular WMH comprehensively. In contrast, the sFazekas scale evaluates the entire cerebral hemisphere as a whole and assigns a single overall grade, thereby simplifying the rating procedure, improving scoring efficiency, and reducing subjectivity associated with regional boundary delineation.

In clinical practice, the reproducibility and scoring efficiency of rating tools form the foundation for their widespread application. When visually assessing WMH, inter-rater agreement is particularly critical, as it directly affects the reliability and comparability of the evaluation results (19). Meanwhile, enhancing rating efficiency contributes to the effective implementation of clinical work. Our study demonstrated that the sFazekas scale exhibited high inter-rater consistency and remained robust across multiple MRI platforms, indicating good stability and broad applicability. Furthermore, the sFazekas scale required less time to complete than the Fazekas scale, which was consistent with our expectations. Moreover, we observed a strong correlation between the sFazekas score and WMH volume, providing robust evidence for the validity and reliability of the sFazekas scale as a measure of WMH burden.

Previous studies have consistently demonstrated that age is a major determinant of WMH, with both the prevalence and volume of WMH increasing markedly with advancing age (20–22). The results of this study suggest that age is a significant predictor of sFazekas score, which is highly consistent with previous findings, indicating that this scoring system has high sensitivity in reflecting age-related WMH burden. Regarding the influence of sex, most studies have not identified significant differences. A previous retrospective study in a Chinese population reported no statistically significant difference in WMH prevalence between males and female (21), and our findings support this conclusion, suggesting that sex is not a major determinant of WMH burden in our cohort. On the other hand, various vascular risk factors, including hypertension (23), diabetes (20), and a history of stroke (24), have been shown to be closely associated with the occurrence of WMH. Our study further confirmed this conclusion. These risk factors may contribute to WMH accumulation by promoting CSVD (25).

We acknowledge that more complex rating systems are valuable for specialists and researchers. The Fazekas scale reflects the spatial distribution of WMH and provides stronger explanatory power for their etiology and clinical associations (17, 26). In contrast, the sFazekas scale simplifies the scoring procedure, reduces assessment time without compromising reliability, and facilitates rapid evaluation of the overall WMH burden. Notably, the sFazekas score showed a stronger correlation with F-DWM (*ρ* = 0.907) than with F-PVWM (*ρ* = 0.739). This difference may be partly explained by the scoring logic of sFazekas, which is closely related to the higher of the F-DWM and F-PVWM grades, as well as by the different score distributions in our cohort. F-PVWM had a substantially higher proportion of grade 0 scores than F-DWM (36.2% vs. 8.3%), whereas the distributions of sFazekas and F-DWM were more similar. Thus, the stronger association with F-DWM may reflect differences in score distribution and scoring characteristics rather than a specific weighting toward deep white matter.

This study has several limitations that should be noted: (i) the study included cerebrovascular patients aged ≥50, and the generalizability to younger populations remains to be investigated. Because our data were collected retrospectively from medical records, migraine and multiple sclerosis histories were not systematically assessed. Although no patients with a documented diagnosis or history of migraine or multiple sclerosis were identified in the final study population, undocumented or previously unrecognized migraine or multiple sclerosis could not be completely excluded, potentially introducing residual confounding. (ii) The study employed a cross-sectional design, reflecting only a single time point, and the utility of the sFazekas scale for monitoring WMH progression needs to be further explored in longitudinal studies; (iii) The sample was derived from two hospital sites within a single institution and included data from only three MRI scanners, which may limit the generalizability of our findings. Future multicenter studies with a broader range of equipment are warranted to enhance the generalizability and robustness of the results. Furthermore, the relatively small proportion of severe WMH may limit the generalizability of our findings to patients with severe WMH burden. Further validation in clinical cohorts with a greater proportion of patients with severe WMH is warranted. (iv) In addition, this study included MRI data acquired using scanners with different field strengths (1.5 T and 3.0 T) and different platforms. Given that WMH volume quantification is influenced by differences in field strength and scanner platform, pooling data acquired from different devices may introduce some variability. (v) Clinical data in this retrospective study were limited to vascular risk factors. Prospective studies incorporating more detailed clinical parameters are necessary to examine how the sFazekas scale relates to additional clinical outcomes and to refine its clinical utility.

## 5 Conclusion

In summary, the simplified Fazekas visual rating scale (sFazekas) demonstrates reliability comparable to the conventional Fazekas scale, with the practical advantage of shorter scoring time, suggesting its potential utility in the clinical assessment of overall white matter hyperintensity burden in patients with cerebral small vessel disease.

## Data Availability

The raw data supporting the conclusions of this article will be made available by the authors, without undue reservation.

## Footnotes

1
https://stnava.github.io/ANTs/

2
https://hub.docker.com/r/wmhchallenge/sysu_media
